# Theaflavins attenuate iron overload-induced liver oxidative injury by inhibiting hepatocyte ferroptosis

**DOI:** 10.7150/ijbs.103971

**Published:** 2025-04-21

**Authors:** Junzhou Chen, Mingdao Mu, Xin Lai, Chen Liu, Yuheng Luo, Jun He, Bing Yu, Quyuan Wang, Huifen Wang, Daiwen Chen, Aimin Wu

**Affiliations:** 1 Institute of Animal Nutrition, Sichuan Agricultural University, Chengdu 611130, China.; 2 Key Laboratory for Animal Disease-resistance Nutrition of China Ministry of Education, Sichuan Agricultural University, Chengdu 611130, China.; 3 School of Medicine, Southeast University, Nanjing 210096, China.

**Keywords:** iron overload, ferroptosis, liver injury, theaflavins, antioxidant

## Abstract

Liver, as a major iron storage organ, is particularly sensitive to oxidative stress-induced damage stemming from iron overload. Thus, antioxidant therapies are often used to reverse oxidative stress-induced tissues damage, however, the cellular mechanisms remain enigmatic. This study investigated the protective effects and mechanisms of theaflavins, a nature production from tea, against oxidative damage in iron overload hepatocytes and mouse liver. Iron overload disrupted iron metabolism in hepatocytes by activating inflammation and enhancing HO-1 expression, leading to hepatic ferroptosis and serious liver damage. Additionally, iron overload inhibited Xc^-^ system (SLC7A11 and SLC3A2), decreasing GSH synthesis, ultimately further induced ferroptosis. Intriguingly, theaflavins supplementation robustly counteracted iron overload-induced ferroptosis and subsequent liver damage. Notably, inhibition of HO-1 and activation of Xc^-^ system provided the mechanistic insights into theaflavins inhibition of hepatocytes ferroptosis. Take together, these results highlight ferroptosis as an inducer of iron overload-induced liver damage, which is inhibited by theaflavins. This nature product form tea represents a potential therapeutic approach to attenuating organ damage in iron overload individuals.

## 1. Introduction

Iron is a vital nutrient that plays a pivotal role in various physiological processes, including oxygen transport, energy metabolism, and cellular proliferation 1, 2. However, an abundance of iron in the body, referred to as iron overload, can result in significant organ damage, especially in the liver 3. The liver is the primary organ responsible for iron storage and regulation, making it highly susceptible to iron overload-induced injury 4. Mounting evidence suggests a close association between iron overload and a wide spectrum of liver diseases, such as hereditary hemochromatosis 3, nonalcoholic fatty liver disease, and hepatitis C virus infection 4. Moreover, patients with chronic liver disease often exhibit disturbed iron homeostasis, further exacerbating the progression of liver injury 5.

Recent years have witnessed substantial advancements in unraveling the molecular processes responsible for liver damage caused by iron overload. One of the key pathways implicated in this process is ferroptosis, a novel form of cell death closely linked with free iron (Fe^2+^)-mediated lipid peroxidation 6. Ferroptosis is triggered by the accumulation of reactive oxygen species (ROS) and lipid peroxidation products, leading to catastrophic cell membrane damage and ultimately cell death 7, 8. Emerging evidence indicates that ferroptosis is intricately linked to various biological processes, including iron metabolism, amino acid and polyunsaturated fatty acid metabolism, and the biosynthesis of glutathione (GSH), NADPH, and coenzyme Q10 9. Disruption of these pathways has been shown to contribute to the onset and progression of ferroptosis 10. Notably, ferroptosis has been implicated in the pathogenesis of several liver disorders associated with iron overload 8. Therefore, targeting ferroptosis represents a promising therapeutic strategy for mitigating iron overload-induced liver injury 11.

Theaflavins is a group of natural polyphenolic compounds derived from black and oolong teas 12. Theaflavins is formed during the fermentation process of tea leaves, where catechins, such as epicatechin and epigallocatechin-3-gallate, undergo oxidation by polyphenol oxidases and peroxidases 13. The resulting theaflavins possesses potent antioxidant properties attributed to their unique structural features 13. Meanwhile, theaflavins commonly found in black tea, are frequently highlighted in scientific literature for their ability to chelate iron ions effectively. This chelation property allows theaflavins to bind to free iron, thereby reducing iron's availability to participate in harmful reactions such as the Fenton reaction, which can lead to oxidative stress and cellular damage 14. Accumulating evidence suggests that theaflavins exerts a wide range of biological activities, including neuroprotective, cardioprotective, nephroprotective and anti-inflammatory, antimicrobial effects 15. These pleiotropic actions of theaflavins are mediated through the modulation of various cellular signaling pathways, such as the activation of nuclear factor erythroid 2-related factor 2 (Nrf2)/Kelch-like ECH-associated protein 1 (Keap1) signaling cascade and the inhibition of mitogen-activated protein kinase (MAPK) signaling 16. While the liver-protective properties of theaflavins have been documented, their capacity to mitigate iron overload-induced liver damage by suppressing ferroptosis remain largely uninvestigated.

This study aimed to explore the protective effects of theaflavins against liver damage caused by iron overload and to elucidate the underlying molecular mechanisms, with a particular emphasis on ferroptosis. Our findings revealed that iron overload triggered liver injury by activating ferroptosis in hepatocytes. Activation of HO-1 and inhibition of system Xc^-^ (SLC3A2 and SLC7A11) provided a mechanistic explanation for this phenomenon. Remarkably, theaflavins demonstrated a pronounced inhibitory effect on ferroptosis by downregulating HO-1 expression and activating system Xc^-^, thereby protecting iron overload-induced liver injury. These findings highlight the therapeutic potential of theaflavins as a natural agent for the prevention and treatment of iron overload-related liver disease.

## 2. Materials and methods

### 2.1. Chemicals and antibodies

**Figure 1:**
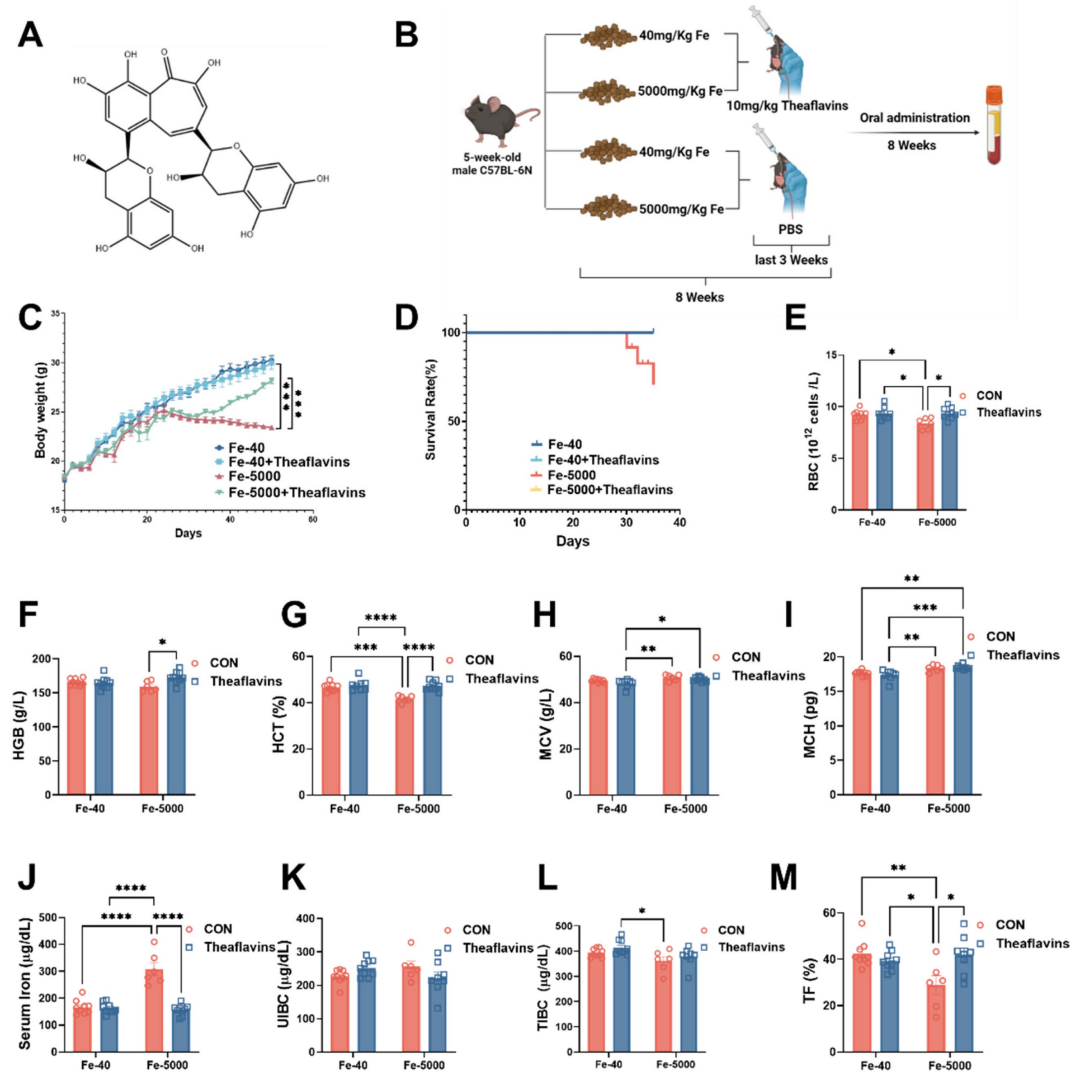
** Effects of theaflavins supplementation on growth performance and blood parameters in mice fed high-iron diets.** (A) Structural formula of theaflavins. (B) Overview of the experimental design and treatment concentrations. (C) Body weight of mice in four groups measured throughout the experiment. Mouse body weight was recorded weekly for the first three weeks and daily after initiating gavage administration. (D) Survival curves of mice at 8 weeks of the experiment. (E) Red blood cell count (RBC). (F) Hemoglobin (HGB). (G) Hematocrit (HCT). (H) Mean corpuscular volume (MCV). (I) Mean corpuscular hemoglobin (MCH). (J) Serum iron level. (K) Unsaturated iron binding capacity (UIBC). (L) Total iron binding capacity (TIBC). (M) Transferrin saturation (TF). Statistical significance is indicated as follows: *P < 0.05, **P < 0.01, ***P < 0.001, ****P < 0.0001.

Theaflavins (purity ≥ 98%) were sourced from Teaturn Bio-pharmaceutical Co., Ltd (Fig. 1A). The addition dosage of theaflavins is based on previous research 17. Primary antibodies targeting TfR1, Fpn, FTH/L, GPX4, SLC7A11 and HO-1 were acquired from Abcam, while β-actin was procured from Santa Cruz Biotechnology.

### 2.2. Animal experiments

The animal experimental protocols were authorized by the Institutional Animal Care and Use Committee of the Laboratory Animal Center at Sichuan Agricultural University (SICAU-2015-033). For this study, 5-week-old male C57BL/6N mice were used and maintained in a controlled setting with a temperature of (21 ± 2) °C, humidity between 45-55%, and a 12-hour light/dark cycle. The mice were randomly divided into a 2*2 factorial arrangement and were provided diets containing either 40 mg/kg or 5000 mg/kg FeSO_4_ 18, 19. Additionally, they were administered either 0 or 10 mg /kg theaflavins via gavage during the final three weeks of the experiment. The duration of the experiment was 8 weeks, after which all mice were euthanized (see Fig. 1B).

### 2.3. Isolation and culture of mouse primary hepatocytes

Mouse primary hepatocytes were isolated and cultured using the following method: liver tissue was digested with collagenase type II (Gibco) to separate the hepatocytes 20, 21. Live hepatocytes were then isolated by centrifugation using Percoll (Sigma-Aldrich). These hepatocytes were plated onto 6-well or 12-well plates that had been pre-coated with collagen. The primary hepatocytes were initially cultured in DMEM medium for 24 hours, after which the wells were washed once with PBS. Fresh DMEM medium was then added, and the cells were exposed to various reagents. Cells were harvested at specific time points for analysis.

### 2.4. Determination of hematological parameters

Whole blood samples were collected into EDTA-coated tubes, and hematological parameters, including red blood cell count (RBC), hemoglobin (HGB), hematocrit (HCT), mean corpuscular volume (MCV), mean corpuscular hemoglobin (MCH) and mean corpuscular hemoglobin concentration (MCHC) were measured using the automatic biochemical analyzer (Shenzhen Myriad Biomedical Electronics Co, China).

### 2.5. Assessment of iron status

Serum iron levels and tissue non-heme iron concentrations were determined. The quantification of non-heme iron content in liver and spleen was conducted using a previously described method 22. Blood samples were analyzed to determine serum iron levels, total iron-binding capacity (TIBC), unsaturated iron-binding capacity (UIBC), and serum transferrin (TF) concentrations using a standardized procedure 1.

### 2.6. ALT, AST and enzyme activity assay

Serum alanine aminotransferase (ALT) and aspartate aminotransferase (AST) activities were measured using an automatic biochemical analyzer to assess liver injury. To assess oxidative stress, malondialdehyde (MDA) and glutathione (GSH) levels were measured using commercially available assay kits from Jiancheng Bioengineering Institute. The assays were performed according to the manufacturer's protocols.

### 2.7. Real-time quantitative PCR (qPCR) and western blot

Hepatic total RNA extraction was carried out by Trizol reagent (Invitrogen) according to the manufacturer 's specifications, and the mRNA diluted to 1 μg/μl. The working solution was prepared following the protocols provided in the Reverse Transcription Kit (Takara). Relative gene expression levels were determined using the ΔΔCT method, calculating cycle threshold (Ct) values and normalizing them to the housekeeping gene *Hprt*. Total protein was extracted from the liver using RIPA buffer supplemented with phenylmethanesulfonylfluoride (PMSF, 1 mM). Western blotting was performed according to a previously established protocol. Primary antibodies were diluted 1:1000, and secondary goat anti-mouse and anti-rabbit antibodies conjugated with horseradish peroxidase (HRP) (Santa Cruz) were diluted 1:3000.

### 2.8. Histological analysis

Liver tissue samples were collected and preserved in 4% paraformaldehyde solution in PBS overnight. After fixation, the tissues were embedded in paraffin. Subsequently, 5-μm thick sections were prepared and stained with hematoxylin and eosin (H&E) as well as Masson's trichrome stain (Masson) for microscopic examination and histological analysis.

### 2.9. Measurement of lipid ROS and total ROS

To assess cellular levels of reactive oxygen species (ROS) and lipid ROS, primary hepatocytes were treated with 50 μM H_2_DCFDA (Sigma-Aldrich) and 5 μM CD11-BODIPY (Invitrogen), respectively, for 30 minutes at 37°C. Following incubation, the cells underwent three washes with PBS. Subsequently, the fluorescence intensity and analysis of cells were performed using flow cytometry.

### 2.10. Determination of labile iron pool (LIP)

FerroFarRedTM (Goryo Chemical) was employed to test the intracellular labile iron (Fe^2+^) of primary hepatocytes following the manufacturer's provided guidelines. Cells were incubated with 1 μM FerroOrange for 30 min at 37°C, washed with PBS, and analyzed by flow cytometry.

### 2.11. *Hmox*1 knockdown

Short hairpin RNAs (shRNAs) targeting mouse Hmox1 (Ho-1-shRNA: AGCCACACAGCACTATGT AAA) were incorporated into the pAdEasy-U6-CMV-mCherry adenovirus vector (HanBio) following the manufacturer's protocol. Recombinant adenovirus amplification was carried out using HEK 293A cells. Primary hepatocytes were seeded in 12-well plates and transduced with *Hmox1*-shRNA adenoviruses for 24 h before any additional treatment.

### 2.12. Adenovirus overexpression of Hmox1

Adenoviruses expressing *Hmox1* (Ad-Hmox1) were purchased from HanBio (4.0×10^10^ PFU/ml). Primary hepatocytes were seeded in 12-well plates and transduced with Ad-Hmox1 for 24 h before any additional treatment.

### 2.13. Statistical analysis

Statistical analyses were performed using GraphPad Prism. All data are presented as means ± standard errors of the means (SEM). Statistical tests comprised unpaired two-tailed Student's t-test and one-way or two-way analysis of variance (ANOVA), as appropriate, followed by Bonferroni post hoc tests. Distribution comparisons were performed using a Kolmogorov-Smirnov test. A p-value less than 0.05 was considered statistically significant. All results were visualized using GraphPad Prism 9.5.0 software.

## 3. Results

### 3.1. Theaflavins supplementation ameliorates growth performance decline and inflammatory anemia in mice fed high-iron diets

The experimental procedure and feeding protocol are shown in Fig. 1B. We found that theaflavins played a crucial role in mitigating the decline in growth performance and inflammatory anemia induced by iron overload. Mice in the iron-overloaded group began to lose weight around the fourth week, and by the eighth week, a significant difference in body weight compared to the control group mice was evident. However, oral administration of 200 µL of 10 mg/kg/day theaflavins significantly restored the weight loss caused by iron overload (Fig. 1C). Notably, the iron overload group had a 30% mortality rate at the end of the experiment, a phenomenon not observed in the other treatment groups (Fig. 1D). Iron overload led to a significant decrease in the levels of RBC, HGB, and HCT, all of which were restored normal levels following theaflavins supplementation. Additionally, the levels of MCV, MCH, and HCT levels showed a significant decrease after theaflavins intervention, while the levels of MCV and MCH did not change significantly (Fig. 1E to I). Iron overload consistently increased serum iron and decreased TIBC and TF% compared to the control group, with these serum iron-related indices significantly returning to normal levels following theaflavins administration (p < 0.01). However, UIBC did not exhibit significant changes (Fig. 1J to M).

### 3.2. Theaflavins administration reduces inflammatory response and attenuates iron overload-induced liver injury

**Figure 2:**
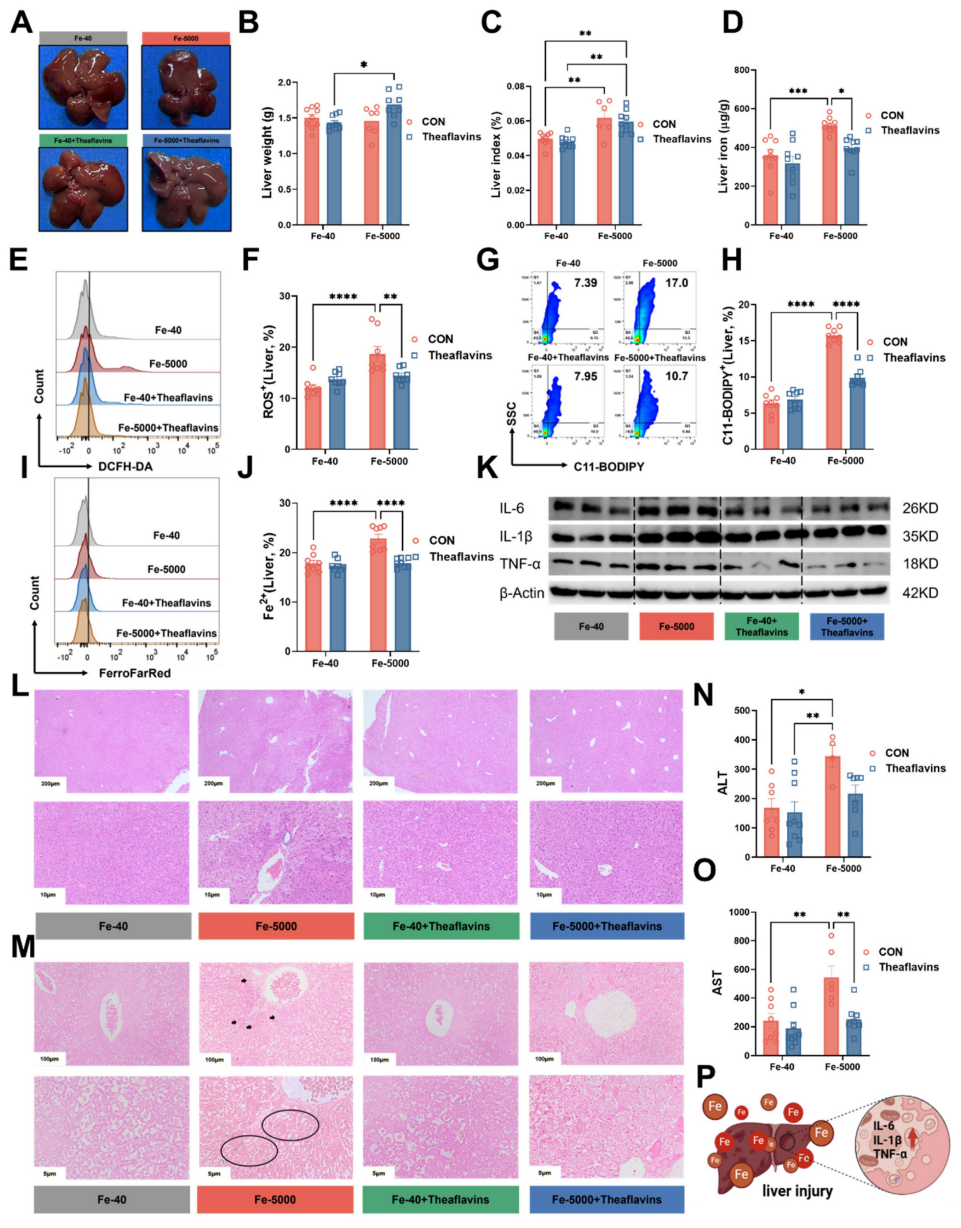
** Prophylactic effect of theaflavins on high iron-induced liver injury.** (A) Gross morphology of intact liver from different treatment groups. (B) Liver weight in different treatment groups. (C) Liver index (liver weight/body weight ratio). (D) Hepatic iron content. (E) Flow cytometry results histogram of total ROS in hepatocytes. (F) Positive rate of total ROS in hepatocytes. (G) Flow cytometry images of lipid peroxidation in hepatocytes. (H) Positive rate of lipid peroxidation in hepatocytes. (I) Flow cytometry histogram of labile iron pool (LIP) in HO-1 knockout hepatocytes. (J) Positive rate of LIP in hepatocytes. (K) Statistical Analysis Western blotting was performed to detect IL-6, IL-1β, and TNF-α protein expression. (L-M) Representative images of hematoxylin and eosin (H&E) staining and Perls staining of mouse liver sections. (N-O) Serum levels of ALT and AST. (P) Overview of Liver injury and inflammatory response. Statistical significance is indicated as follows: *P < 0.05, **P < 0.01, ***P < 0.001, ****P < 0.0001.

Iron overload typically results in liver injury 23. To evaluate the impact of theaflavins on the iron overload-induced liver injury, serum samples and liver tissues were collected for analysis. Mice fed high iron (Fe-5000) diets exhibited a significant increase in liver color, liver index and iron content (Fig. 2A, C and D), but without impact on liver weight (Fig. 2B). Meanwhile, the flow cytometry results revealed a dramatic increase in the levels of C11-BODIPY (a marker of ferroptosis), ROS, and Fe2+ in iron-overloaded primary hepatocytes (Fig. 2E-J). However, theaflavins supplementation significantly decreased C11-BODIPY, ROS, and Fe^2+^ levels, which further confirmed that theaflavins is an inhibitor of ferroptosis (Fig. 2E-J). Iron overload robustly induced hepatic tissue inflammation by upregulating *IL-6, IL-1β,* and *TNF-α* mRNA expression, while theaflavins supplementation remarkably attenuated iron overload induced hepatic tissue inflammation by downregulating *IL-6, IL-1β,* and *TNF-α* mRNA expression (Fig. S1A-C). Similar results were observed in the protein expression levels of IL-6, IL-1β*,* and TNF-α (Fig. 2K Fig. S1D-F). Furthermore, hematoxylin and eosin (H&E) staining Perls staining and Masson's trichrome staining of the liver tissues revealed that iron overload induced liver injury and fibrosis (Fig. 2L and M Fig. S1G), findings further supported by elevated serum levels of alanine aminotransferase (ALT) and aspartate aminotransferase (AST) (Fig. 2N and O). Interestingly, theaflavins administration significantly mitigated iron overload-induced liver injury (Fig. 2L-O). Briefly, high-dose iron leads to hepatic tissue inflammation and injury in mice, while theaflavins addition effectively alleviates iron overload-induced liver inflammation and injury (Fig. 2P).

### 3.3. Theaflavins supplementation inhibits hepatic ferroptosis induced by iron overload in vivo

**Figure 3:**
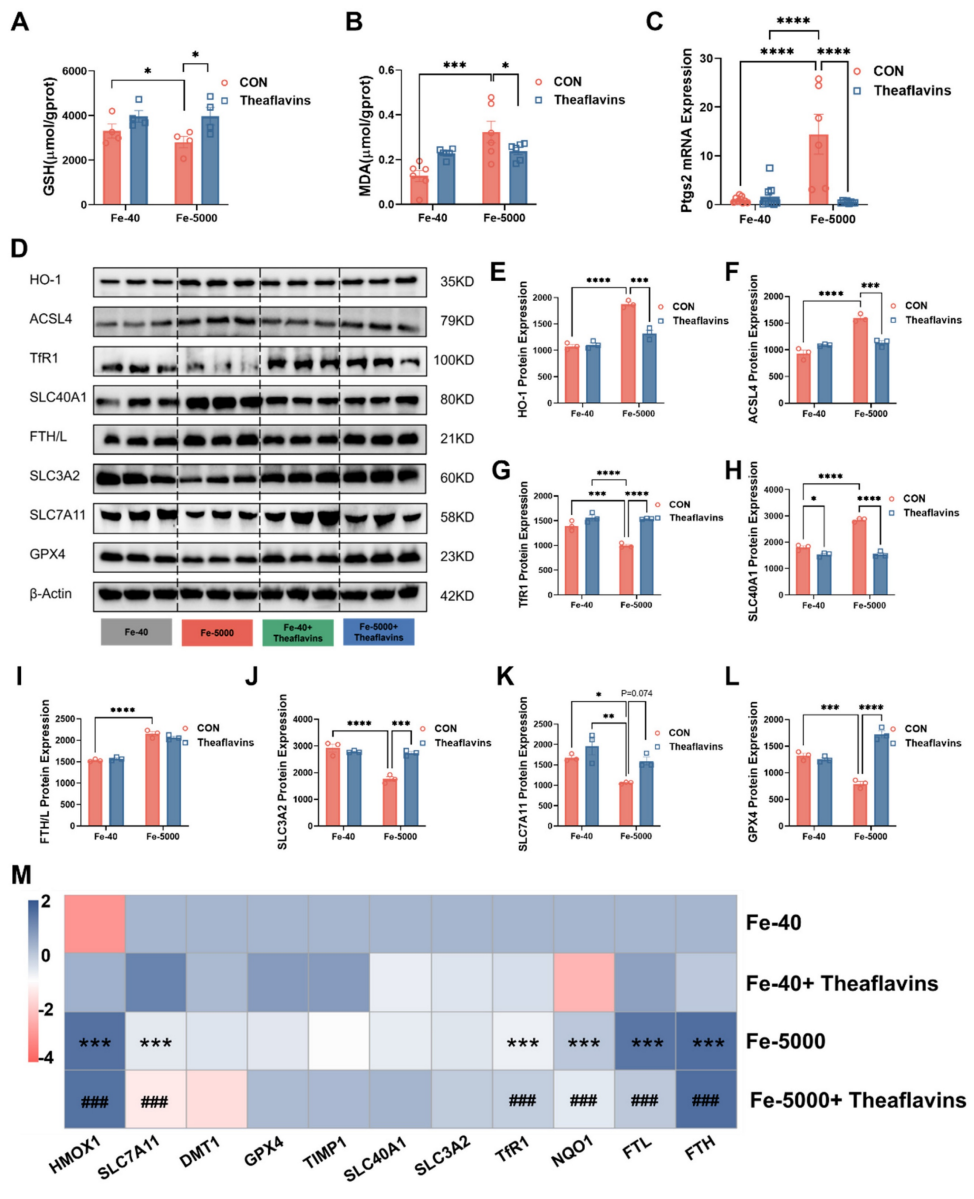
** Theaflavins alleviate high-iron induced liver injury by inhibiting the occurrence of ferroptosis in cells.** (A) GSH level in the liver. (B) MDA level in the liver. (C) mRNA expression of Ptgs2. (D) Western blot analysis of ferroptosis-related protein expression. (E-L) Quantification of HO-1, ACSL4, TfR1, SLC40A1, FTH/L, SLC3A2, SLC7A11and GPX4 protein expression. (M) Heatmap of correlation analysis between treatment groups and the genes of ferroptosis. Statistical significance is indicated as follows: *P < 0.05, **P < 0.01, ***P < 0.001, ****P < 0.0001.

Ferroptosis is a complex process of cell death triggered by iron-dependent lipid peroxidation 6, 23. The consequences of this process are profound, leading to changes in cellular biochemistry measurable through indicators like glutathione (GSH) and malondialdehyde (MDA) levels 24. In this study, iron overload markedly reduced GSH contents while elevating MDA levels in mice liver. However, both indicators were significantly restored after theaflavins treatment (p < 0.01), suggesting theaflavins as a potential an inhibitor of ferroptosis (Fig. 3A-B). Simultaneously, an increase in *Ptgs2* (a marker gene for ferroptosis) gene expression was observed in the livers of mice in the iron overload group, which was significantly reduced by the intervention of theaflavins (Fig. 3C). To further investigate this phenomenon, the expression of key genes and pathways involved in ferroptosis was analyzed in the livers of mice with iron overload, with or without theaflavins treatment. The results showed that iron overload increased the mRNA and protein expression of HO-1. Notably, this increase was normalized after theaflavins intervention (Fig. 3D, E and M). A similar result was observed in the mRNA and protein expression of acyl-CoA synthetase long-chain family member 4 (ACSL4), a ferroptosis activator that shapes cellular lipid composition (Fig. 3D and F). Iron overload also significantly increased the protein and gene expression of FTH/L and SLC40A1 in the iron metabolic pathway, which was rescued by theaflavins, indicating theaflavins' ability to inhibit ferroptosis by restoring iron metabolism (Fig. 3D, H-I, M). Iron overload significantly down-regulated the gene expression of TfR1, while the addition of theaflavins restored the expression of TfR1 without significant changes in protein levels (Fig. 3D, G, M). Meanwhile, while the protein levels of SLC7A11 remained unchanged, the expression of SLC3A2 and GPX4 was inhibited by iron overload, with theaflavins showing therapeutic potential (Fig. 3D, J-L, M). Furthermore, the expression of *TIMP1* genes was elevated under iron overload, further suggesting that the cells may undergo iron metamorphosis (Fig. 3M). Interestingly, the levels of NCOA4 and LC3II proteins were higher in the iron overload group than in the control group, suggesting that ferritinophagy may play an important role in hepatocytes ferroptosis mediated by iron overload. However, the protein expression of NCOA4 and LC3II return to normal after theaflavins administration (Fig. S2A-C).

### 3.4. Theaflavins inhibits iron overload-induced ferroptosis in primary hepatocytes

**Figure 4:**
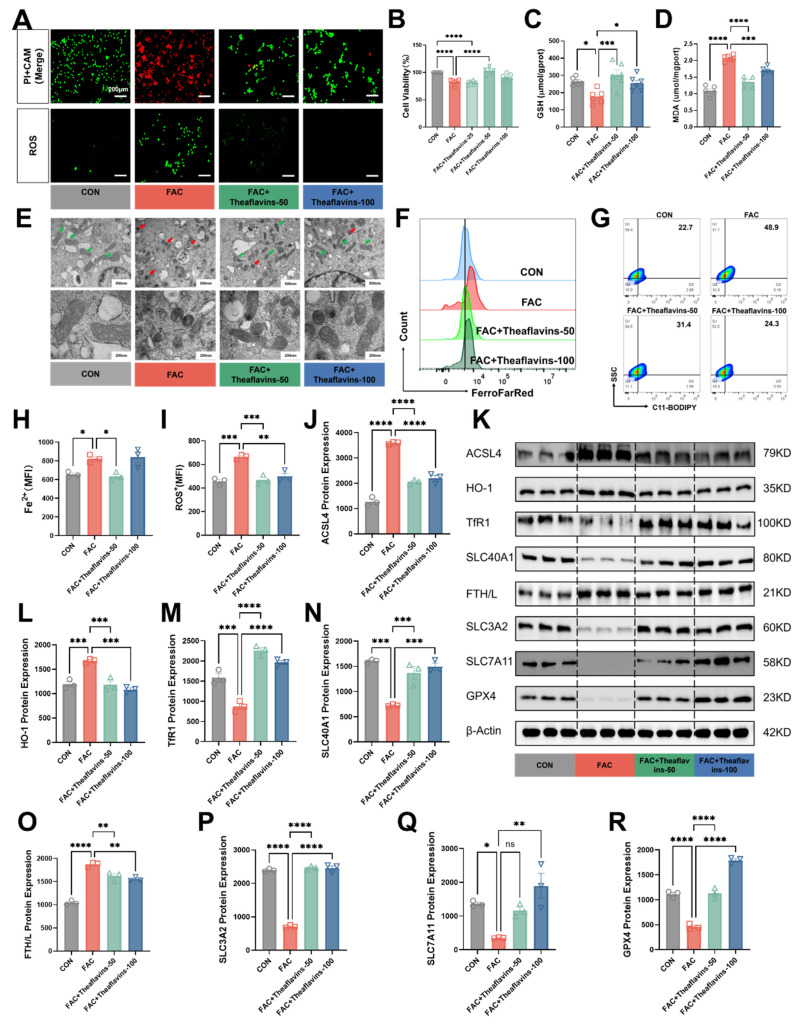
** Inhibition of primary hepatocytes ferroptosis by theaflavins.** (A) Fluorescently labeled PI+CAM and ROS (200μm). (B) Cell viability assessed by Cell Counting Kit-8 (CCK-8) assay. (C) GSH levels in hepatocytes. (D) MDA levels in hepatocytes. (E) Transmission electron microscopy results (500nm and 200nm). (F) Flow cytometry histogram of labile iron pool (LIP) in hepatocytes. (G) Flow cytometry results images of lipid peroxidation in hepatocytes. (H) Mean fluorescence intensity (MFI) of LIP in hepatocytes. (I) MFI of total ROS in hepatocyte. (J-R) Western blot analysis and quantification of ferroptosis-related and antioxidant-related protein expression: (J) ACSL4, (K) Western blot analysis of ferroptosis-related protein expression. (L) HO-1, (M) TfR1, (N) SLC3A2, (O) FTH/L, (P) SLC40A1, (Q) SLC7A11, and (R) GPX4. Statistical significance is indicated as follows: *P < 0.05, **P < 0.01, ***P < 0.001, ****P < 0.0001.

To further clarify the role of theaflavins in ferroptosis induced by iron overload, Ferric ammonium citrate (FAC) at a concentration of 1000 µM was added to the culture medium of primary hepatocytes, followed by the addition of 50 μM and 100 μM theaflavins. Initially, cell viability and cell death of FAC-treated primary hepatocytes were assessed using CAM/PI staining and CCK8 kit. As s shown in Fig. 4A and 4B, treatment with 1000 μΜ FAC markedly decreased the cell viability and increased cell death in primary hepatocytes. However, theaflavins supplementation significantly attenuated high does FAC-induced hepatocyte cell death (Fig. 4A and B). Moreover, FAC treatment decreased GSH content but increased MDA levels in primary hepatocytes, indicating iron overload may induce ferroptosis. Conversely, theaflavins supplementation increased GSH content and decreased MDA levels (Fig. 4C and D). Subsequently, the mitochondrial morphology in the FAC-treated group underwent changes characteristic of ferroptosis, with mitochondria becoming smaller and rounder and cristae beginning to disappear (Fig. 4E). Interestingly, the mitochondrial morphology of the group treated with theaflavins was gradually restored to normal characteristics, further confirming the inhibitory effect of theaflavins on iron overload-induced ferroptosis (Fig. 4E). Meanwhile, the flow cytometry results revealed a dramatic increase in the levels of C11-BODIPY (a maker of ferroptosis), ROS and Fe^2+^ in FAC-treated primary hepatocytes (Fig. 4A, F-I). However, theaflavins supplementation significantly decreased C11-BODIPY, ROS and Fe^2+^ level, which further confirmed theaflavins is an inhibitor of ferroptosis (Fig. 4A, F-I). Next, protein expression analysis showed that FAC treatment induced ferroptosis in primary hepatocytes, evidenced by significant changes in the expression of major ferroptosis pathway related proteins (Fig. 4K and S2). Specifically, TfR1, GPX4, SLC7A11, SLC3A2 and SLC40A1 were significantly downregulated, while FTH/L, ACSL4, HO-1, NCOA4 and LC3II were upregulated (Fig. 4J-R, S2D-F). We repeated this experiment using RSL3 as a model of ferroptosis and obtained similar results (Fig. S3). Therefore, these results indicate theaflavins is an inhibitor of ferroptosis.

### 3.5. Theaflavins supplementation inhibits hepatocyte ferroptosis by downregulating HO-1

**Figure 5:**
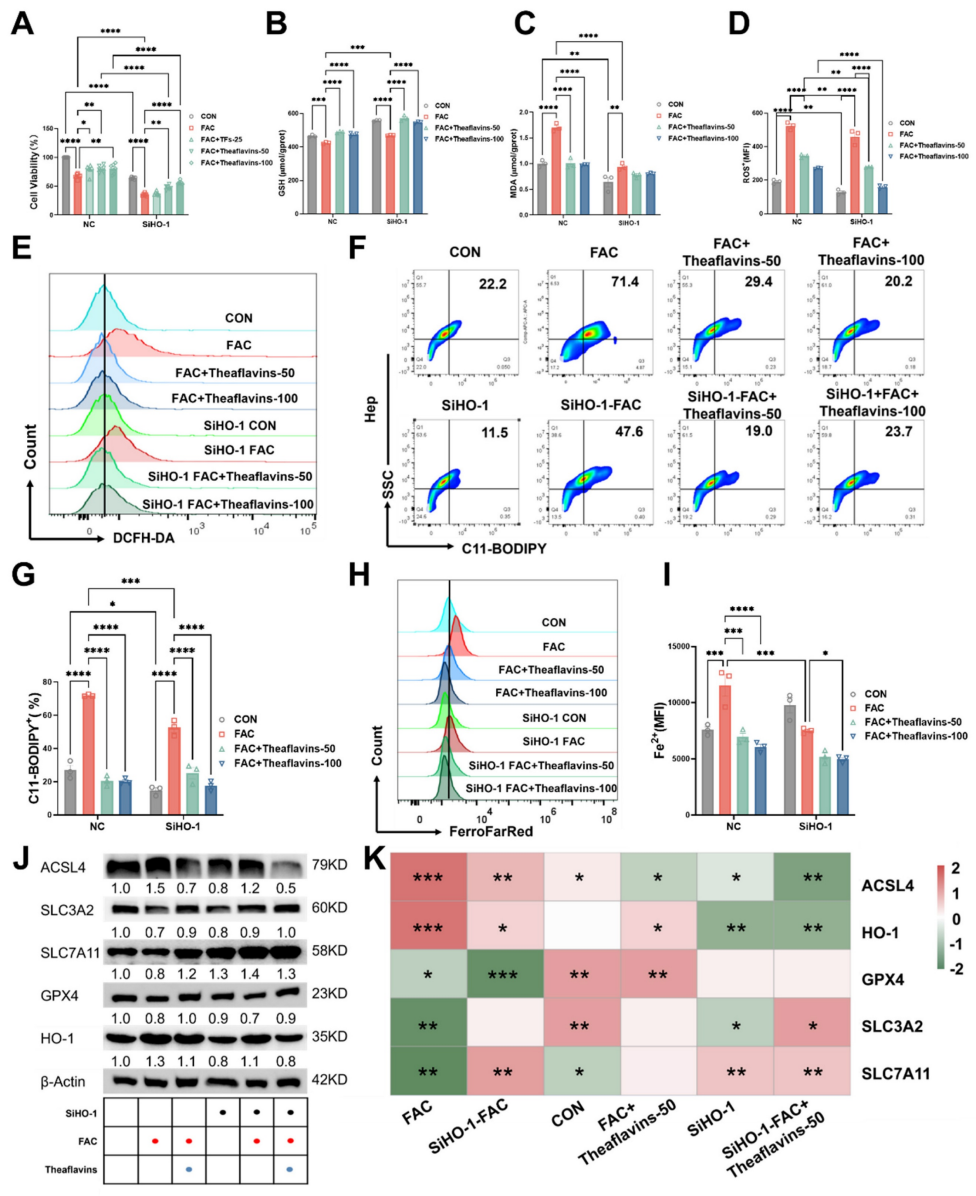
** The effects of knockdown of HO-1 on inhibition of ferroptosis by theaflavins.** (A) Cell viability assessed by Cell Counting Kit-8 (CCK-8) assay. (B) GSH levels in HO-1 knockout hepatocytes. (C) MDA levels in HO-1 knockout hepatocytes. (D) MFI of total ROS in HO-1 knockout hepatocytes. (E) Flow cytometry results histogram of total ROS in HO-1 knockout hepatocytes. (F) Flow cytometry images of lipid peroxidation in HO-1 knockout hepatocytes. (G) Positive rate of lipid peroxidation in HO-1 knockout hepatocytes. (H) Flow cytometry histogram of labile iron pool (LIP) in HO-1 knockout hepatocytes. (I) MFI of LIP in HO-1 knockout hepatocytes. (J) Western blot analysis of antioxidant pathway-related protein expression in HO-1 knockout hepatocytes. (K) Heatmap of correlation analysis between treatment groups and proteins related to the ferroptosis pathway.

Our prior research revealed that HO-1 exhibits a dual role in ferroptosis and ensuing acute iron overload-induced liver injury resulting from intraperitoneal administration of iron dextran. While it demonstrates certain protective capabilities, its excessive activation can significantly contribute to ferroptosis^1^. This study on significant diet-induced chronic liver damage highlights the need for further exploration of the connection between heme oxygenase-1 (HO-1) and ferroptosis. To further investigate the role of HO-1 in the suppression of ferroptosis mediated by theaflavins, HO-1 was silenced in primary hepatocytes using HO-1 shRNA. In HO-1 knockdown hepatocytes, cell viability was significantly reduced, with a more pronounced decline observed following FAC treatment. However, theaflavin intervention effectively restored cell viability, indicating its protective role (Fig. 5A). Interestingly, knocking down HO-1 resulted in a significant increase in glutathione (GSH) levels, indicating enhanced cellular antioxidant capacity. Conversely, the decrease in malondialdehyde (MDA) levels suggested a reduction in lipid peroxidation (Fig. 5B and C). Notably, FAC treatment, which induces oxidative stress, did not result in significant changes in the HO-1 knockdown cells, indicating that the knockdown HO-1conferred resistance to FAC-induced oxidative damage. This protective effect was further supported by lower ROS, C11-BODIPY, and Fe^2+^ levels in the FAC-treated HO-1 knockdown cells compared to FAC-treated normal cells (Fig. 5D-I). All the three indexes showed a significant decrease after theaflavins intervention, with the effect of the theaflavins-treated group being more pronounced in the HO-1 knockdown cells compared to normal cells (Fig. 5D-I). Interestingly, knockdown of HO-1 significantly reduced ACSL4 protein expression in the FAC-treated group compared to the normal FAC-treated group, thereby preventing ACSL4 from catalyzing cellular lipid peroxidation to inhibit ferroptosis, and up-regulated the expression of SLC3A2 and SLC7A11 proteins, while GPX4 protein did not change significantly (Fig. 5J-K). Most notably, HO-1 expression was significantly decreased in the FAC-treated group after HO-1 knockdown, with a continued decreasing trend after theaflavins treatment (Fig. 5J-K). Similarly, the same decrease in GSH and increase in MDA after knockdown of HO-1 was observed under the RSL3 ferroptosis model. The promotion of ferroptosis by HO-1 was also further confirmed by flow cytometry results (Fig. S4).

**Figure 6:**
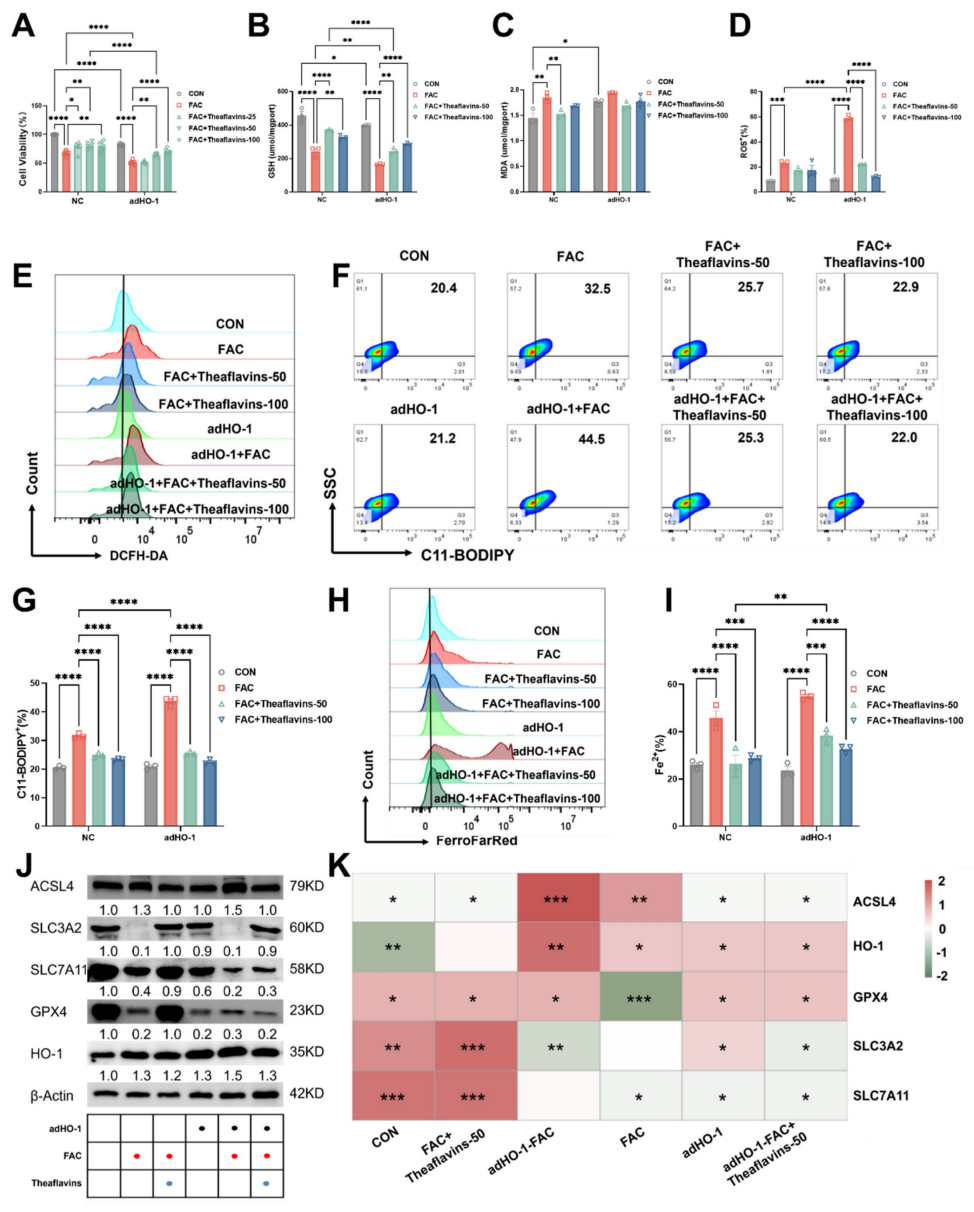
** Effect of HO-1 overexpression on the inhibition of cellular ferroptosis by theaflavins.** (A) Cell viability assessed by Cell Counting Kit-8 (CCK-8) assay. (B) GSH levels in HO-1 overexpressed hepatocytes. (C) MDA levels in HO-1 overexpressed hepatocytes. (D) Positive rate of total ROS in HO-1 overexpressed hepatocytes. (E) Flow cytometry histogram of total reactive oxygen species (ROS) in HO-1 overexpressed hepatocytes. (F) Flow cytometry images of lipid peroxidation in HO-1 overexpressing hepatocytes. (G) Positive rate of lipid peroxidation in HO-1 overexpressed hepatocytes. (H) Flow cytometry results histogram of labile iron pool (LIP) in HO-1 overexpressed hepatocytes. (I) Positive rate of LIP in HO-1 overexpressed hepatocytes. (J) Expression level of proteins related to antioxidant pathway. (K) Heatmap of correlation analysis between treatment groups and proteins related to the ferroptosis pathway. Statistical significance is indicated as follows: *P < 0.05, **P < 0.01, ***P < 0.001, ****P < 0.0001.

Subsequently, HO-1 was overexpressed in primary hepatocytes using adenovirus-HO-1 (Fig. 6). Primary hepatocytes with HO-1 overexpression were treated with FAC and theaflavins for 6 hours, and ferroptosis was assessed by measuring LIP, ROS, and C11-BODIPY levels. In hepatocytes with HO-1 overexpression, cell viability was markedly impaired, and this effect was further exacerbated by FAC treatment. Theaflavin intervention, however, significantly ameliorated the loss of cell viability (Fig. 6A).

In contrast to HO-1 knockdown, HO-1 overexpression significantly upregulated ferroptosis-related indexes, such as Fe^2+^, ROS, C11-BODIPY and MDA levels, but decreased GSH content (Fig. 6B-I), indicating HO-1 overexpression further activated ferroptosis in hepatocytes. Interestingly, theaflavins supplementation effectively mitigated ferroptosis induced by HO-1 expression, decreasing the levels of Fe^2+^, ROS, C11-BODIPY and MDA, but increasing GSH content in primary hepatocytes (Fig. 6B-I). Consistent with our previous findings, theaflavins upregulated the expression of SLC3A2 protein in primary hepatocytes overexpressing HO-1, while downregulating the expression of ACSL4, SLC7A11, and GPX4 (Fig. 6J-K). The same results were corroborated in the RSL3 model, confirming the mechanism by which theaflavins inhibit cellular ferroptosis by inhibiting HO-1 (Fig. S5). Taken together, our findings suggest that theaflavins suppress iron overload-induced ferroptosis in hepatocytes by downregulating heme oxygenase-1 (HO-1) expression.

## 4. Discussion

Iron, an essential mineral for the human body, plays a crucial role in various biological processes. Unlike other nutrients, the body lacks an active pathway for iron excretion, meaning that any excess iron consumed is stored within the body 25, 26, which can lead to serious diseases, including organ failure 26-28. The liver and heart are particularly vulnerable to iron overload. The liver is essential for maintaining iron equilibrium within the body. Consequently, any impairment of liver function, particularly in individuals with chronic liver disease, can lead to substantial alterations in iron regulation 3, 29. Iron-induced damage is mainly caused by its interaction with ROS. This interaction generates hydroxyl radicals, highly reactive species that can induce lipid peroxidation, oxidation of amino acid residues, DNA strand scission, and protein degradation. Recent studies suggest that ferroptosis plays a crucial role in the pathogenesis of acute liver failure induced by acetaminophen (APAP) overdose 30. Another study mentioned that patients with iron overload-induced liver injury had significantly increased levels of oxidative stress, and that the damage caused by iron overload could be alleviated through the use of ferroptosis inhibitors 31. These findings suggest that the regulation of cellular ferroptosis is an important target for the treatment of liver diseases, including iron overload-induced liver injury.

Our previous studies and those of others have found that iron overload causes liver injury and liver fibrosis by inducing ROS accumulation and activating ferroptosis in hepatocytes 32. In the present study, we found that high doses of iron decreased the survival rate of mice after 8 weeks of feeding, which may be related to high-iron induced liver injury as well as other internal organ lesions 33. Additionally, iron overload was observed to result in a significant increase in IL-6, TNF-α and IL-1β protein expression in mice. These cytokines are key elements of the inflammatory cascade response, and their elevated expression suggests that iron overload may exacerbate the inflammatory response in vivo 33. Iron metabolism is closely related to inflammation. Hepcidin, a key regulator of systemic iron metabolism, is stimulated by the cytokine IL-6. As a result, hepcidin is classified as an acute-phase protein, produced and released by the liver in response to inflammation. During inflammatory conditions, systemic iron balance is disrupted, typically causing hypoferremia or a reduction in serum iron levels, as our research confirms 34. Ferroptosis occurs when excess iron accumulates in hepatocytes, leading to a surge in ROS production. These reactive oxygen species, in turn, activate lipid peroxidation, a key step leading to ferroptosis. At the same time, iron overload leads to the upregulation of HO-1 and a massive breakdown of heme (hemoglobin) 35. The massive breakdown of heme further triggers the release of Fe^2+^. These Fe^2+^ accumulate within the cell and cause toxic effects on the cell. Excess Fe^2+^ lead to a disruption of the redox balance within the cell, which in turn triggers cellular ferroptosis. GSH is a key antioxidant that scavenges intracellular free radicals and maintains cellular redox balance. In the presence of iron overload, the function of system Xc^-^ is inhibited, leading to a decrease in the synthesis of GSH 36, 37. This decrease in GSH weakens the body's antioxidant capacity, making hepatocytes more susceptible to damage from oxidative stress 38 1. This oxidative stress not only exacerbates the cytotoxicity induced by iron overload, but also further increases the sensitivity of hepatocytes to ferroptosis. The present study builds on these findings and explores in greater detail the intricate relationship between iron overload, liver injury and ferroptosis.

Previous studies have explored the mechanisms of iron-induced cell death in mice and found that iron overload can lead to ferroptosis. Iron homeostasis is a delicate balance maintained by several key proteins such as TfR1, SLC40A1 and FTH/L, which are essential for cellular health. Once iron overload disrupts this balance, it triggers a cascade of events leading to cellular dysfunction and death. In this case, the inflammatory response is a key factor, closely linked to the molecular triggers of ferroptosis. GPX4, a glutathione peroxidase, plays a key role in attenuating the inflammatory response and preventing ferroptosis 39. Its down-regulation in the presence of iron overload indicates a compromised antioxidant defense system. Similarly, SLC7A11 and SLC3A2, which are involved in amino acid transport and glutathione synthesis, respectively, were also down-regulated, further suggesting that the antioxidant mechanism is compromised 40, 41. Iron overload also upregulated the expression of HO-1, ACSL4, TfR1, PTGS2, and NQO1 proteins. These findings suggest that iron overload promotes iron transport and disrupts the antioxidant system 42, 43.

Theaflavins interventions are effective in restoring such changes. By scavenging harmful ROS and suppressing inflammatory signaling pathways, theaflavins may help mitigate the damage caused by iron overload 44. Theaflavins successfully mitigated the negative impact of elevated iron levels on serum iron and erythropoiesis. Studies have shown that although polyphenols reduce iron absorption through their anti-inflammatory properties, they can still indirectly promote erythropoiesis by modulating inflammatory responses and improving the microenvironment, thereby demonstrating a positive effect on the body's hematopoietic function 45. The research observed that theaflavins bind to iron with high affinity, preventing its uncontrolled accumulation in the bloodstream. This binding not only stabilizes iron levels but also ensures its efficient delivery to erythroid precursor cells for hemoglobin synthesis 46. Surprisingly, theaflavins appeared to alleviate liver injury due to iron overload by inhibiting cellular ferroptosis through decreasing the expression of HO-1. In this study, we performed HO-1 knockdown and overexpression in hepatocytes to further validate this result. This observation suggests that theaflavins may act by inhibiting HO-1 expression while activating system Xc^-^, thereby mitigating the deleterious effects of iron overload 47. Up-regulation of HO-1 levels in the cell is a hallmark of oxidative stress, with downstream effects especially in the pro-oxidative state 48, 49. By targeting HO-1, theaflavins can modulate iron metabolism and mitigate iron-induced cell death. Future studies will aim to further elucidate the molecular mechanisms underlying these observations and explore the potential clinical applications of theaflavins in the treatment of iron-overload related disorders.

## 5. Conclusion

This research revealed that excessive iron accumulation in mice disrupts iron balance and the antioxidant defense system. This disturbance led to ferroptosis in the liver, causing oxidative damage and inflammatory anemia. Theaflavins can effectively attenuate iron overload-induced oxidative damage and ferroptosis by suppressing HO-1 expression while activating system Xc^-^. These results underscore the promising therapeutic potential of theaflavins in alleviating diseases associated with iron metabolism disorders.

## Supplementary Material

Supplementary figures.

## Abbreviations

ALT: alanine transaminase
AST: aspartate transaminase
DMT1: divalent metal transporter 1
RBC: red blood cell count
FAC: ferric citrate
FTH/L: ferritin heavy/light chain
Fpn: ferroportin
GSH: glutathione
GSSG: glutathione, Oxydized
GPX4: glutathione peroxidase 4
HGB: hemoglobin
HCT: hematocrit
HO-1: heme oxygenase 1
MCV: mean corpuscular volume
MCH: mean corpuscular hemoglobin
MCHC: mean corpuscular hemoglobin concentration
MDA: malondialdehyde
NOQ1: quinone oxidoreductase 1
Ptgs2: Prostaglandin G/H synthase 2
ROS: reactive oxygen species
SLC3A2: solute carrier family 3 member 2
SLC7A11: solute carrier family 7 member 11
TF: transferrin saturation
TIBC: total iron binding capacity
TfR1: transferrin receptor 1
UIBC: unsaturated iron binding capacity
