# Supplementary material for: Theaflavins attenuate iron overload-induced liver oxidative injury by inhibiting hepatocyte ferroptosis
Source: Int J Biol Sci. 2025 Apr 21;21(7):3030–44. doi: 10.7150/ijbs.103971 (PMC12080394; doi:10.7150/ijbs.103971)
Supplement: Supplementary file 1 — Supplementary figures. [file ijbsv21p3030s1.pdf]

Figure S1

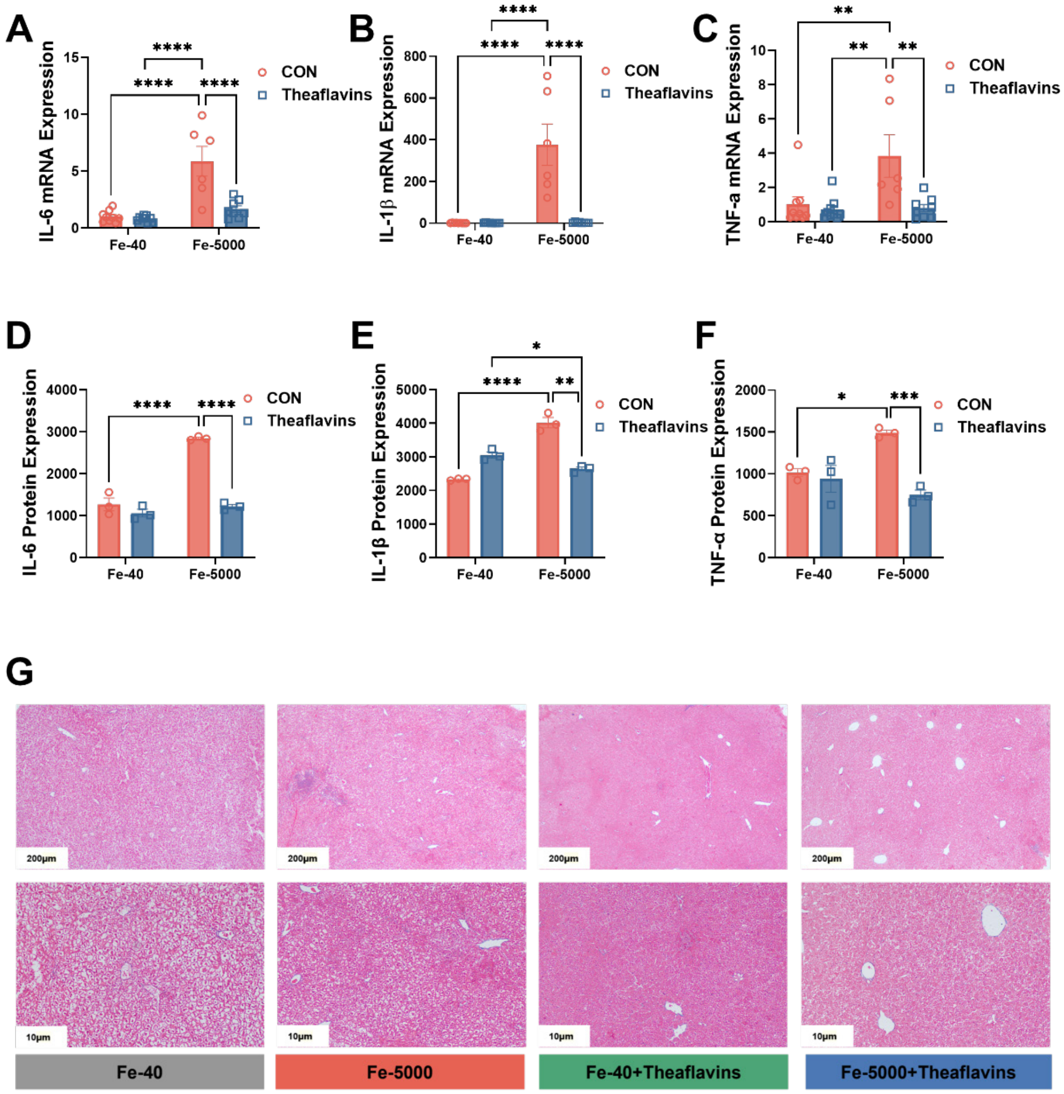

Fig. S1. Hepatic inflammatory markers and fibrosis. (A-C) Quantification of IL-6, IL-1β, and TNF-α in the mouse livers from each group. (D-F) Statistical Analysis Western blotting was performed to detect IL-6, IL-1β, and TNF-α protein expression. (G) The Masson's trichrome staining sections of mouse liver tissue(200 μm and 10 μm). Statistical significance is indicated as follows: $^*P < 0.05$, $^{**}P < 0.01$, $^{***}P < 0.001$, $^{****}P < 0.0001$.

Figure S2

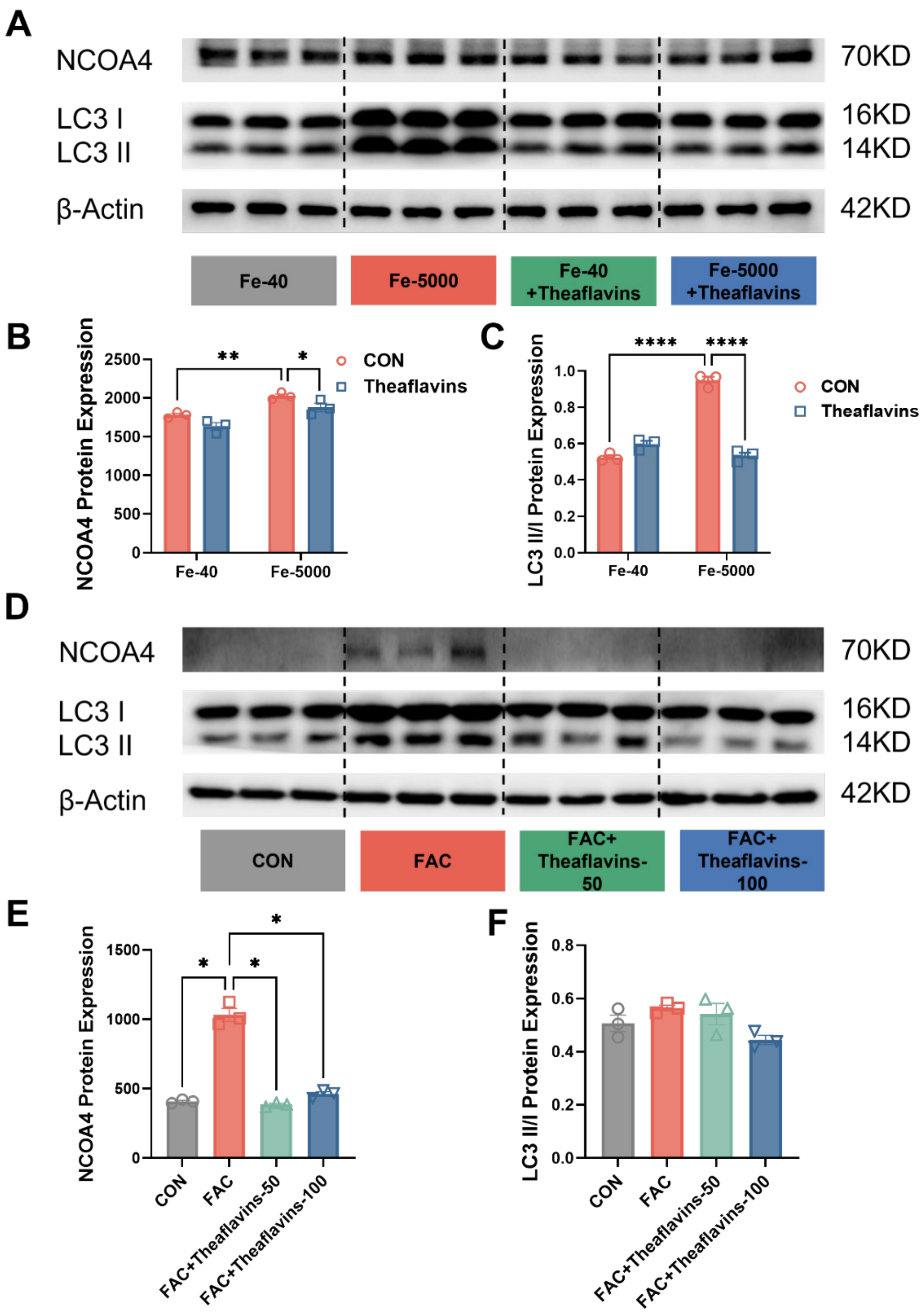

Fig. S2. Iron autophagy in mouse liver tissue and hepatocytes. (A) Expression level of proteins related to iron autophagy pathway in liver. (B and C) Quantification of NCOA4 and LC3 I/II protein expression. (D) Expression level of proteins related to iron autophagy pathway in hepatocytes. (E and F) Quantification of NCOA4 and LC3 I/II protein expression

in hepatocytes. Statistical significance is indicated as follows: *P < 0.05, **P < 0.01, ***P < 0.001, ****P < 0.0001.

Figure S3

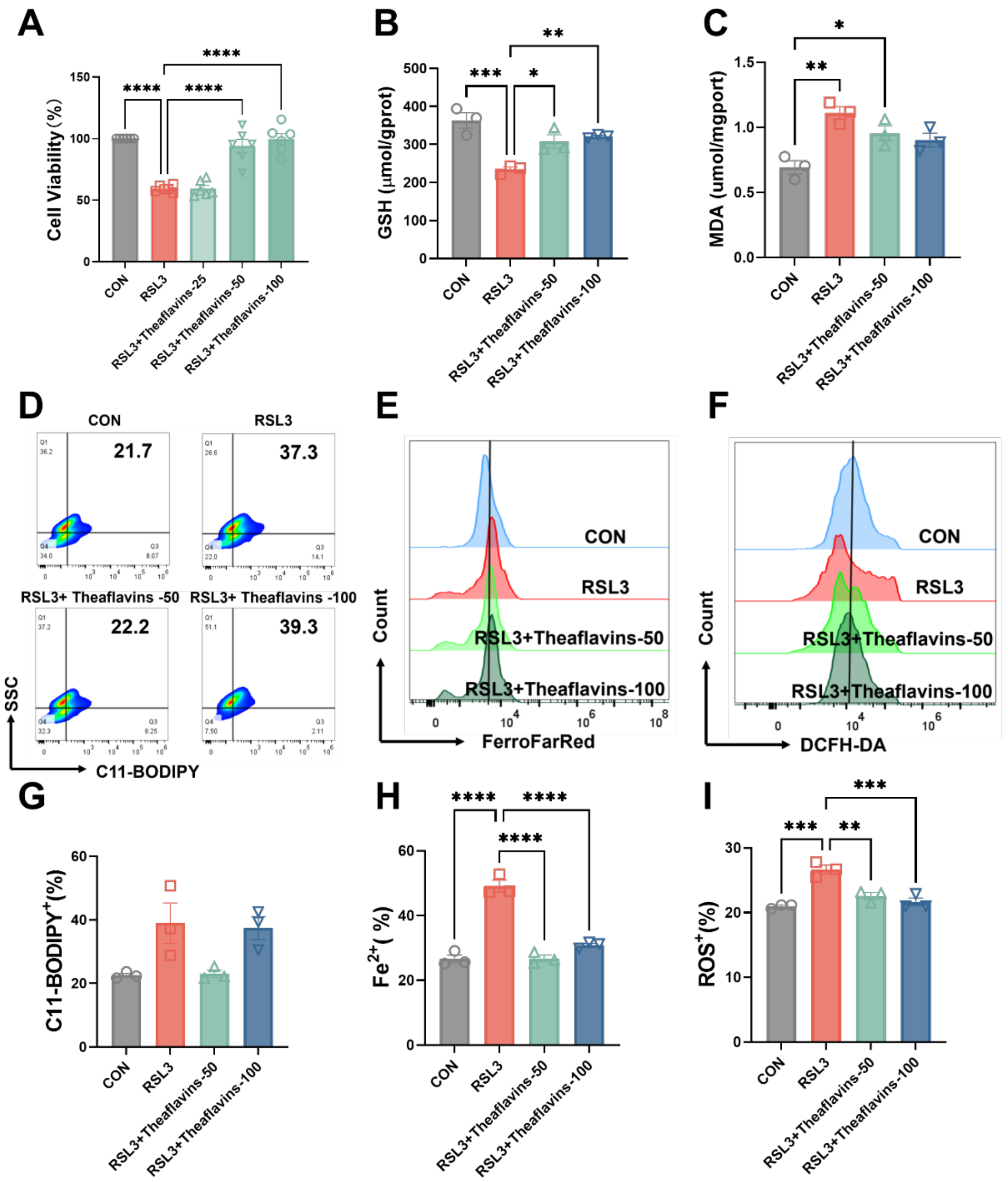

Fig. S3. Inhibition of primary hepatocytes ferroptosis by theaflavins. (A) Cell Counting Kit-8. (B) GSH levels in hepatocytes. (C) MDA levels in hepatocytes. (D) Flow cytometry results images of lipid peroxidation in hepatocytes. (E) Flow cytometry results histogram of labile iron pool (LIP) in hepatocytes. (F) Flow cytometry results histogram of total reactive oxygen species (ROS) in hepatocytes. (G) Positive rate of lipid peroxidation in hepatocytes. (H) Positive rate of LIP in hepatocytes. (I) Positive rate of total ROS in hepatocytes. Statistical significance is indicated as follows: $*P < 0.05$, $**P < 0.01$, $***P < 0.001$, $****P < 0.0001$.

Figure S4

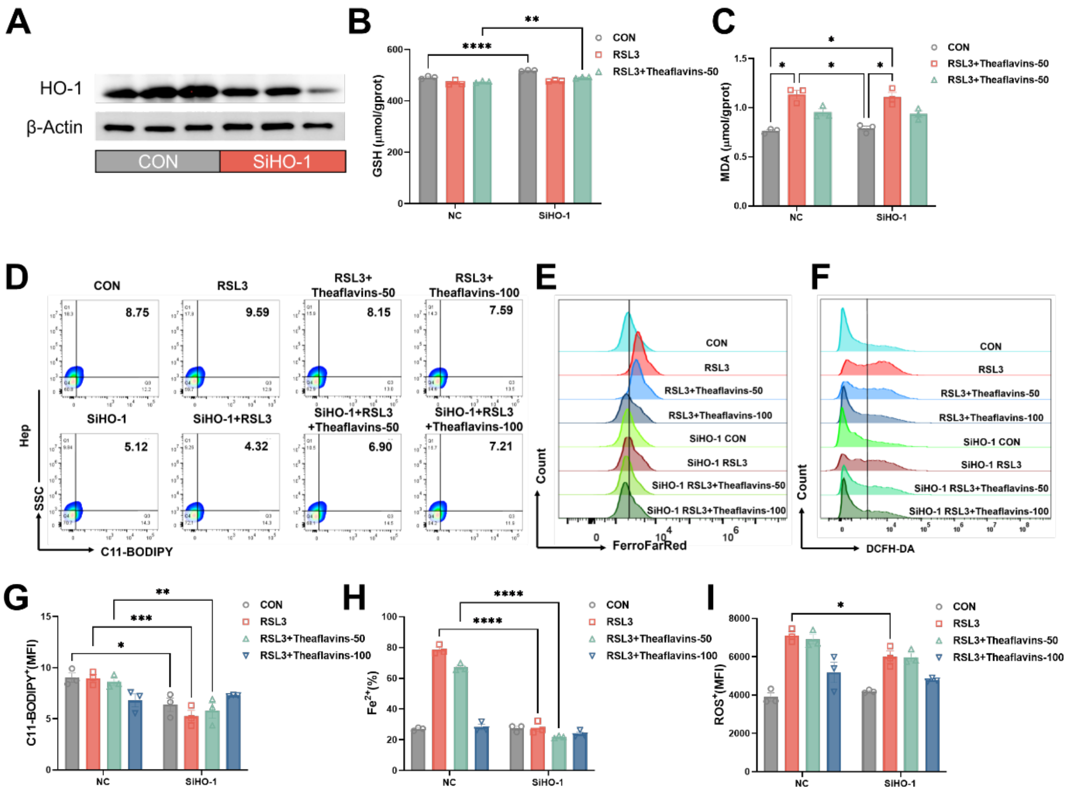

Fig. S4. The effects of knockdown of HO-1 on inhibition of ferroptosis by theaflavins. (A) Knockdown of HO-1 in hepatocytes reduces HO-1 protein expression. (B) GSH levels in HO-1 knockout hepatocytes. (C) MDA levels in HO-1 knockout hepatocytes. (D) Flow cytometry images of lipid peroxidation in HO-1 overexpressed hepatocytes. (E) Flow cytometry results histogram of labile iron pool (LIP) in HO-1 knockout hepatocytes. (F) Flow cytometry histogram of total reactive oxygen species (ROS) in HO-1 knockout hepatocytes. (G) MFI of lipid peroxidation in HO-1 knockout hepatocytes. (H) Positive rate of LIP in HO-1 knockout hepatocytes. (I) MFI of total ROS in HO-1 knockout hepatocytes. Statistical significance is indicated as follows: *P < 0.05, **P < 0.01, ***P < 0.001, ****P < 0.0001.

Figure S5

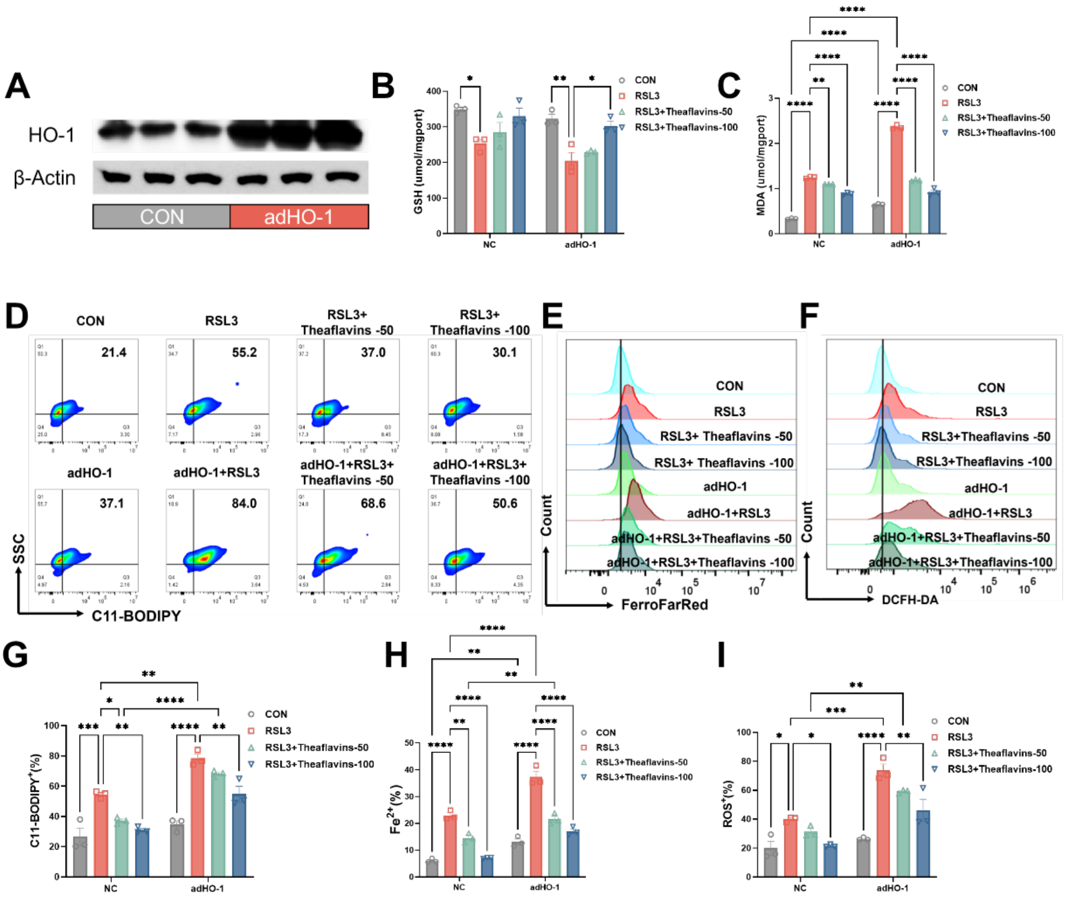

Fig. S5. Effect of HO-1 overexpression on the inhibition of cellular ferroptosis by theaflavins. (A) Overexpression of HO-1 in hepatocytes reduces HO-1 protein expression. (B) GSH levels in HO-1 overexpressed hepatocytes. (C) MDA levels in HO-1 overexpressed hepatocytes. (D) Flow cytometry images of lipid peroxidation in HO-1 overexpressed hepatocytes. (E) Flow cytometry results histogram of labile iron pool (LIP) in HO-1 overexpressed hepatocytes. (F) Flow cytometry histogram of total reactive oxygen species (ROS) in HO-1 overexpressed hepatocytes. (G) Positive rate of lipid peroxidation in HO-1 overexpressed hepatocytes. (H) Positive rate of LIP in HO-1 overexpressed hepatocytes. (I) Positive rate of total ROS in HO-1 overexpressed hepatocytes. Statistical significance is indicated as follows: *P < 0.05, **P < 0.01, ***P < 0.001, ****P < 0.0001.
